# Association between impaired dynamic cerebral autoregulation and BBB disruption in reversible cerebral vasoconstriction syndrome

**DOI:** 10.1186/s10194-023-01694-y

**Published:** 2023-12-19

**Authors:** Yu-Hsiang Ling, Nai-Fang Chi, Li-Ling Hope Pan, Yen-Feng Wang, Chia-Hung Wu, Jiing-Feng Lirng, Jong-Ling Fuh, Shuu-Jiun Wang, Shih-Pin Chen

**Affiliations:** 1 Department of Neurology, Neurological Institute, Taipei Veterans General Hospital, No. 201, Section 2, Shipai Road, Beitou District, Taipei, Taiwan; 2 College of Medicine, National Yang Ming Chiao Tung University, No. 155, Sec. 2, Linong St, Beitou Dist, Taipei, Taiwan; 3 Brain Research Center, National Yang Ming Chiao Tung University, No. 155, Sec. 2, Linong St, Beitou Dist, Taipei, Taiwan; 4 Department of Radiology, Taipei Veterans General Hospital, No. 201, Section 2, Shipai Road, Beitou District, Taipei, Taiwan; 5 Institute of Clinical Medicine, National Yang Ming Chiao Tung University, No. 155, Sec. 2, Linong St, Beitou Dist, Taipei, Taiwan; 6 Department of Medical Research, Taipei Veterans General Hospital, No. 201, Section 2, Shipai Road, Beitou District, Taipei, Taiwan

**Keywords:** Vasospasm, Thunderclap headache, Reversible cerebral vasoconstriction syndrome, Blood-brain barrier, Breakdown, Dynamic, Cerebral autoregulation

## Abstract

**Background:**

Half of the sufferers of reversible cerebral vasoconstriction syndrome (RCVS) exhibit imaging-proven blood-brain barrier disruption. The pathogenesis of blood-brain barrier disruption in RCVS remains unclear and mechanism-specific intervention is lacking. We speculated that cerebrovascular dysregulation might be associated with blood-brain barrier disruption in RCVS. Hence, we aimed to evaluate whether the dynamic cerebral autoregulation is altered in patients with RCVS and could be associated with blood-brain barrier disruption.

**Methods:**

A cross-sectional study was conducted from 2019 to 2021 at headache clinics of a national tertiary medical center. Dynamic cerebral autoregulation was evaluated in all participants. The capacity of the dynamic cerebral autoregulation to damp the systemic hemodynamic changes, i.e., phase shift and gain between the cerebral blood flow and blood pressure waveforms in the very-low- and low-frequency bands were calculated by transfer function analysis. The mean flow correlation index was also calculated. Patients with RCVS received 3-dimensional isotropic contrast-enhanced T2 fluid-attenuated inversion recovery imaging to visualize blood-brain barrier disruption.

**Results:**

Forty-five patients with RCVS (41.9 ± 9.8 years old, 29 females) and 45 matched healthy controls (41.4 ± 12.5 years old, 29 females) completed the study. Nineteen of the patients had blood-brain barrier disruption. Compared to healthy controls, patients with RCVS had poorer dynamic cerebral autoregulation, indicated by higher gain in very-low-frequency band (left: 1.6 ± 0.7, *p* = 0.001; right: 1.5 ± 0.7, *p* = 0.003; healthy controls: 1.1 ± 0.4) and higher mean flow correlation index (left: 0.39 ± 0.20, *p* = 0.040; right: 0.40 ± 0.18, *p* = 0.017; healthy controls: 0.31 ± 0.17). Moreover, patients with RCVS with blood-brain barrier disruption had worse dynamic cerebral autoregulation, as compared to those without blood-brain barrier disruption, by having less phase shift in very-low- and low-frequency bands, and higher mean flow correlation index.

**Conclusions:**

Dysfunctional dynamic cerebral autoregulation was observed in patients with RCVS, particularly in those with blood-brain barrier disruption. These findings suggest that impaired cerebral autoregulation plays a pivotal role in RCVS pathophysiology and may be relevant to complications associated with blood-brain barrier disruption by impaired capacity of maintaining stable cerebral blood flow under fluctuating blood pressure.

**Graphical Abstract:**

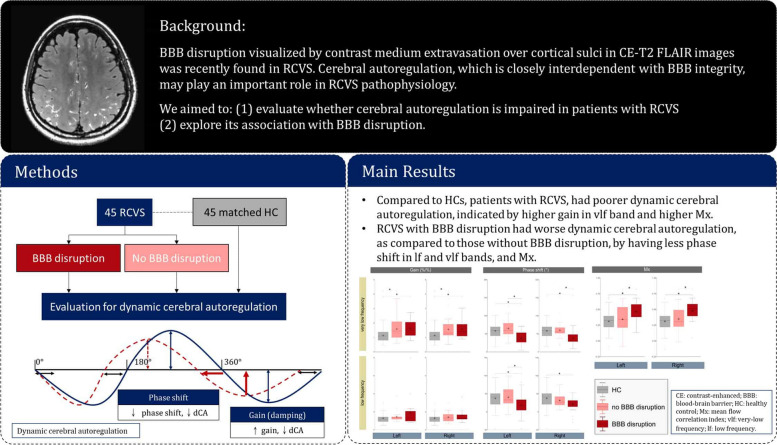

## Background

 Reversible cerebral vasoconstriction syndrome (RCVS) is a characteristic disease known for recurrent thunderclap headaches (TCHs) that are usually elicited by specific triggers, for examples, bath, cough, emotional changes, exertion, straining, and sexual orgasm [1]. RCVS may also be precipitated with vasoactive medications or substances, or pregnancy, postpartum status, other physical stress, or rare causes that altered sympathetic tone such as pheochromocytoma [2–4]. RCVS is potentially fatal since it may be complicated with ischemic stroke, intracranial hemorrhage, convexity subarachnoid hemorrhage, and posterior reversible encephalopathy syndrome [3, 5–7]. Chronic headache lingered after RCVS onset was also reported [8–10]. Neuroimaging examinations are essential in RCVS diagnosis. Cerebral vasoconstriction can be visualized by magnetic resonance angiography (MRA) during the acute phase of RCVS, and its reversibility can be demonstrated by serial studies, usually three months after the first TCH onset [11]. By applying contrast-enhanced fluid-attenuated inversion recovery imaging (CE-FLAIR) imaging, Lee et al. [12] discovered blood-brain barrier (BBB) disruption in RCVS by exhibiting gadolinium-based contrast medium extravasation over the cortical sulci. The finding was later validated, [13] and the temporal profile of imaging-proven BBB disruption was well investigated in an international collaborative study [14]. In RCVS, imaging-proven BBB disruption is most prevalent within the first two weeks of disease onset, preceding the worst time point of vasoconstriction. The chronological order suggests that BBB disruption may play a pivotal role early in RCVS pathogenesis, making vasoconstriction of major cerebral arteries rather a consequence than a cause. Unraveling the potential contributors of BBB disruption in RCVS may lead us to understanding its pathogenesis.

The BBB separates the blood stream and the brain microenvironment. As the essential element of BBB, the endothelial cells form the tight junction as the anatomical barrier, as well as regulate the exchange of ions, nutrients, and metabolites selectively [15, 16]. BBB integrity is crucial for the neurovascular unit functioning. A disrupted BBB may be the result of overwhelmed cerebral autoregulation. Vice versa, when BBB is compromised, it may worsen cerebral autoregulation dysfunction [17]. Therefore, it is rational to speculate that cerebral dysautoregulation plays an important role in RCVS pathophysiology. However, direct evidence demonstrating cerebrovascular dysregulation in RCVS is scarce.

Cerebral autoregulation is an essential mechanism to regulate cerebral vascular tone, ensuring constant cerebral blood flow (CBF) despite the fluctuation of cerebral perfusion pressure (CPP) changes. The concept of dynamic cerebral autoregulation (dCA) is that CBF can maintain stable under rapid CPP changes on the scale of seconds. dCA is well studied in healthy and diseased populations, e.g., elderly, patients with hypertension, dementia, and stroke [18–20]. Research focusing on dCA in ischemic stroke is noteworthy. Studies have indicated that dCA is impaired in acute stroke, [21, 22] and it predicts the prognosis of stroke [23–25] as well as poststroke cognitive impairments [26]. Cerebral autoregulation was applied to generate a personalized and optimized blood pressure target in stroke patients who underwent mechanical thrombectomy [27]. Hence, we deemed dCA a feasible tool to investigate the potential pathogenesis of RCVS. The primary objective of this study is to evaluate whether dCA is impaired in patients with RCVS and to explore its association with BBB disruption.

## Methods

### Ethics

The study protocol was approved by the Institutional Review Board of Taipei Veterans General Hospital (TVGH; TVGH-IRB No 2019-02-013 A & 2020-07-001BC). All participants provided written informed consent before entering the study. All clinical investigations were conducted according to the principles expressed in the Declaration of Helsinki. The corresponding authors have full access to all data in the study and have final responsibility for the decision to submit the research for publication.

### Participants and clinical settings

This prospective study recruited patients with RCVS from the headache center of Taipei Veterans General Hospital, a 3,131-bed national tertiary medical center. Patients newly diagnosed with the acute phase of RCVS within the study period (from September 2019 to November 2021) were approached consecutively. The diagnosis of RCVS was made according to our previously proposed criteria, [28, 29] which was consistent with the criteria proposed in the International Classification of Headache Disorders, third edition (ICHD-3, code 6.7.3) [30]. All the examinations were performed within two days the patients were first seen, including neuroimaging (either computed tomography angiography, CTA, or MRA), transcranial Doppler color-coded sonography, and laboratory tests. Clinical information was collected, including triggers for TCH, premorbid migraine, hypertension, menopausal status, neurological complications, and blood pressure (BP) surge. BP surge was defined as previously reported [14]: systolic BP > 160 mmHg or > 30 mmHg higher than baseline during headache attacks either at the clinic, the emergency department, or the ward, measuring with standard sphygmomanometers. We evaluated dCA and BBB disruption in patients agreed to participate our study (details provided in the following texts). In instances where clinical settings allow, we aimed to minimize the time difference between MRI scan and dCA evaluation. Of note, patients with complications of RCVS, including ischemic stroke, intracranial hemorrhage, convexity subarachnoid hemorrhage, and posterior reversible encephalopathy syndrome, were excluded from the present study. The justification for the exclusion of these individuals from our study was that the observed parameters surrogating dCA were inherently subjected to the influence of concurrent complications. By excluding patients with complications of RCVS, we focused on the association between dCA and RCVS per se.

Healthy controls (HCs), matched by age and sex, were recruited from nearby neighbourhoods and university. Thorough screening was conducted before enrollment to exclude participants with hemodynamically significant atherosclerosis, uncontrolled hypertension, atrial fibrillation, ischemic stroke, intracranial hemorrhage, dementia, and any history of psychiatric or neurological disorders or moderate to severe headaches. HCs with history of using any illicit drugs were also excluded. There was no consanguinity between all HCs and RCVS patients. All HCs received evaluation for dCA. MRI to detect BBB disruption was ***not*** performed in the HCs.

### Evaluations for BBB disruption and dynamic cerebral autoregulation

#### BBB disruption

**Fig. 1:**
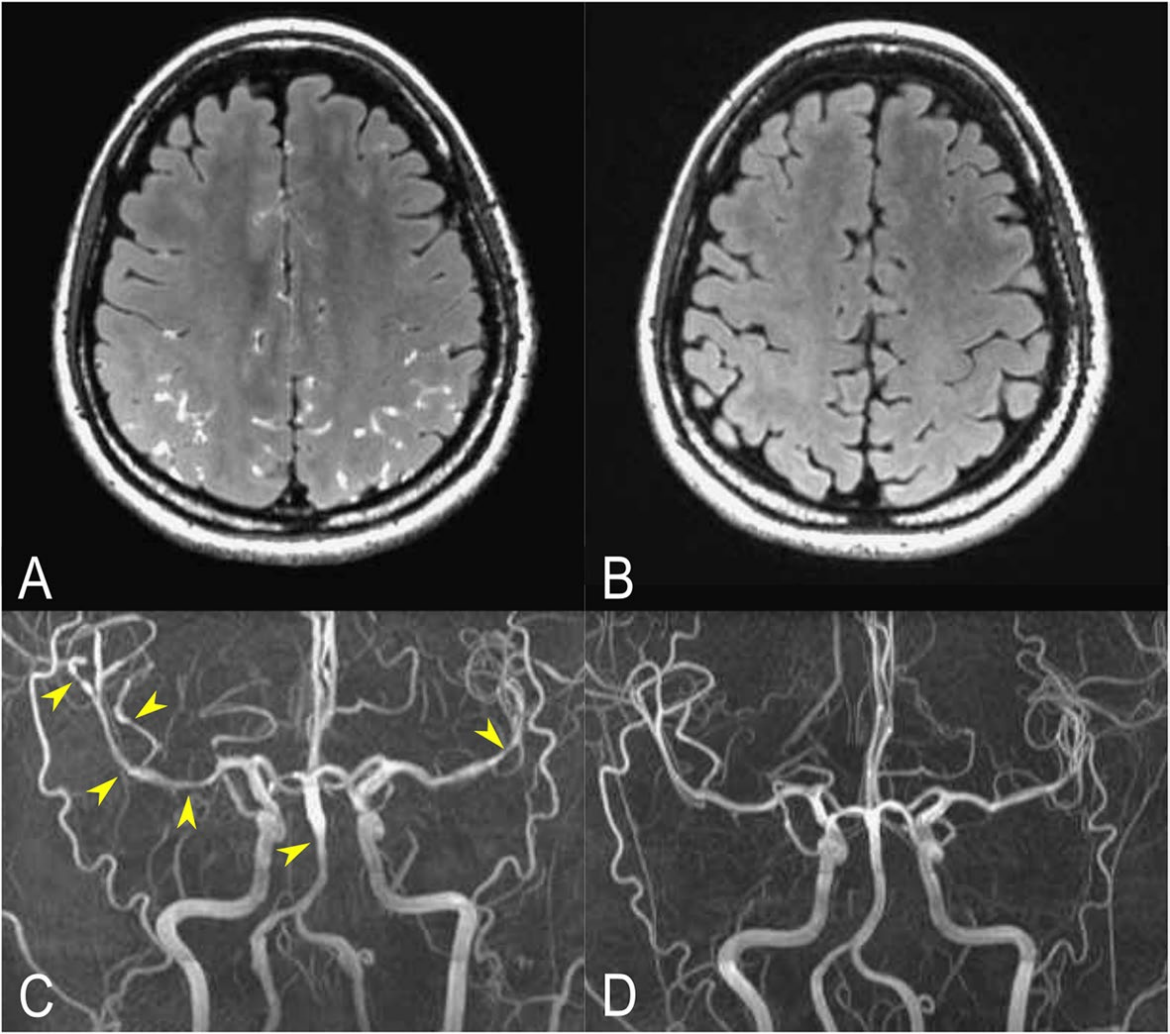
Segmentation of Blood-Brain Barrier Disruption in Patients with Reversible Cerebral Vasoconstriction Syndrome.  **A**  BBB disruption demonstrated by 3-dimensional contrast-enhanced T2-weighted-Fluid-Attenuated Inversion Recovery (FLAIR) image. **B**  Resolution of BBB disruption at three-month follow-up. **C** Vasoconstrictions observed by magnetic resonance angiography. **D**  Recovery of vasoconstrictions at three-month follow-up

All recruited participants with RCVS received 3-dimensional (3D) isotropic contrast-enhanced T2 FLAIR imaging, a validated neuroimaging technique that visualizes BBB disruption by revealing gadolinium (Gd) contrast medium extravasation and its accumulation in the sulci [13, 31]. The MRI protocol is herein concisely reported as follows: The Gd-based contrast agent, gadobutrol, was administered intravenously with the dosage of 0.2mmol/kg (0.2ml/kg; 1mmol/ml was equivalent to 604.72 mg/ml). The isotropic 3D T2-FLAIR sequencing was performed on a 3T MRI machine (MR750; GE Healthcare, Milwaukee, USA), with a 1 mm slice thickness before and after Gd administration. Nine minutes after the administration of Gd, we acquired sagittal 3D CE T2-FLAIR imaging with a fast spin-echo sequence with inversion recovery preparation and variable refocusing flip angles, with parameters set at a repetition time/echo time/inversion time of 6,000/128/1870 milliseconds and a slice thickness of 1 mm. Additionally, the images were reconstructed in axial and coronal planes. Pre-contrast and post-contrast T1-weighted imaging (T1WI) were carried out using two pulse sequences: 3D-T1-ultrasfast gradient echo (repetition time/echo time/inversion time = 9.18/3.68/450 milliseconds, 1 mm section thickness) and 3D-T1-turbo spin echo (repetition time/echo time = 600/12.98 milliseconds, 1 mm section thickness) [13]. Fig. 1 shows vasoconstrictions (Fig. 1 (C)) along with the characteristic appearance of BBB disruption in RCVS on contrast-enhanced FLAIR imaging (Fig. 1 (A)). Both BBB disruption and vasoconstriction recovered at 3-month follow-up MRI (Fig. 1 (B)& (D)). The images were interpreted by two neuroradiologists independently (C.H. Wu and J.F. Lirng). Both radiologists were blinded from clinical information. The final results were based on the consensus of the two neuroradiologists if discrepancy existed.

#### Dynamic cerebral autoregulation

**Fig. 2:**
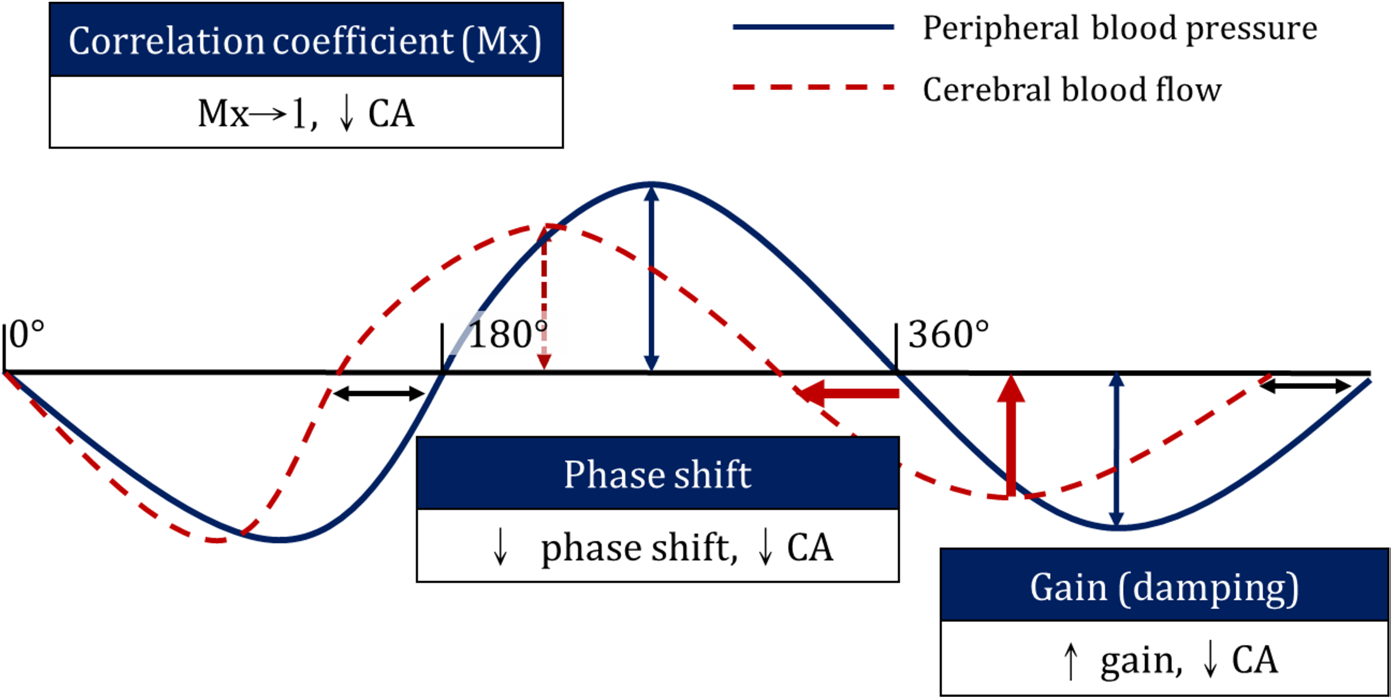
Parameters of dynamic cerebral autoregulation (dCA)

As in our previous studies, [26, 32, 33] the spontaneous fluctuation of peripheral blood pressure (BP) and cerebral blood flow velocity (CBFV) were concomitantly recorded as the surrogates of CPP and CBF respectively. CBFV was recorded using a Doppler sonography monitor (DWL Doppler-Box X, Compumedics DWL, Singen, Germany), BP was recorded using a finger plethysmography (CNAP monitor, CNSystems, Graz, Austria). The recording was in supine and resting state for 5 min. Instead of middle cerebral artery (MCA), CBFV of ***extracranial*** internal carotid artery (ICA) was recorded in avoidance of the poor transtemporal window. The technique was validated in our previous report [33]. Participants with hemodynamically significant atherosclerosis, especially in carotid arteries, were excluded in avoidance of the measurements were confounded by stenotic flow. We applied transfer function analysis (TFA; the MATLAB code is available at http://www.car-net.org/content/resources) to calculate phase shift, gain, and coherence between the CBFV and BP waveforms in the very-low frequency (VLF, 0.02–0.07 Hz) and low frequency (LF, 0.07–0.20 Hz) bands. Concisely, dCA serves to minimize the CBFV fluctuation resulted from BP changes. Therefore, with properly functioning dCA, CBFV demonstrates smaller amplitude as BP fluctuates (small gain), and CBFV returns to baseline earlier than BP (large phase shift, CBFV ahead of BP). On the contrary, one with impaired dCA would have large gain and small phase shift. We also calculated the mean flow correlation index (Mx), which represents the correlation coefficient between the BP and CBFV waveforms under their spontaneous fluctuations [34]. With the Mx approaching 1, the CBFV fluctuates passively to the changes of BP, which indicates impaired dCA; with the Mx approach 0, the CBFV fluctuates independently from the changes of BP, implying a normal functioning dCA. The meaning of abovementioned parameters were summarized in Fig. 2. N.F. Chi, who was blinded from clinical information, was accounted for the calculation and generation of abovementioned parameters. Following previously reported protocols, the average value from bilateral dCA metrics in the HCs was calculated [26, 32]. Each dCA metrics in HCs was compared with metrics derived from both sides in RCVS separately since the vessels in RCVS may be asymmetrically involved and it was impractical to define a “lesion side” in RCVS.

With properly functioning dCA, CBFV demonstrates smaller amplitude as BP fluctuates (small gain), and CBFV returns to baseline earlier than BP (large phase shift, CBFV ahead of BP). The mean flow correlation index (Mx), was not generated with transfer function analysis. It represents the correlation coefficient between the BP and CBFV waveforms. When approaching 1, the CBFV fluctuates passively to the changes of BP, which indicates impaired dCA; when approaching 0, the CBFV fluctuates independently from the changes of BP, implying a normal functioning dCA.

### Treatment and follow-ups

Acute treatment for RCVS (nimodipine in either oral or intravenous form) was given after the diagnostic examinations were completed [29]. All patients were followed at our headache clinics with the interval of every three months on average (but variations were allowed according to clinical necessity), until the resolution of clinical symptoms and the reversibility of vasoconstriction was demonstrated by serial MRA and transcranial Doppler color-coded sonography.

### Statistical analysis

The SPSS version 22.0 (IBM, Armonk, NY, USA) was used for all statistical analyses. Kolmogorov-Smirnov tests were used to examine the normal distribution. Independent sample *t* tests or Mann-Whitney *U* tests were used to compare the differences in continuous variables between RCVS and HC or between RCVS with and without BBB disruption. Chi squared or Fisher’s Exact tests were used to compared differences in the categorical variables between RCVS and HC or between RCVS with and without BBB disruption. The ANOVA with post-hoc Fisher’s least significant difference (LSD) tests or Kruskal-Wallis test with post-hoc Dunn’s multiple comparisons test with Benjamini-Hochberg procedure were used to compare the differences in continuous variables between HC, RCVS without BBB disruption, and RCVS with BBB disruption. Univariate logistic regression was conducted to determine the odds ratio of the presence of BBB disruption related to dCA metrics and the clinical characteristics that showed difference between patients with or without BBB disruption (*p* < 0.1). Significant dCA metrics identified with univariate logistic regression models were included in the multivariable logistic regression after controlling for significant clinical features. When determining sample size, our estimation was based on a previous study [33] that demonstrated the significant differences of dCA between patients with acute ischemic stroke and normal population since there was no previous dCA study available for RCVS. With the statistical power of 80% and a two-tailed significance level of 0.05, the sample size was estimated as 45 for each group. Cohen’s *d* or Cohen’s *f* was used to determine the effect size of the comparisons. All the data were presented as means and standard deviations or as percentage. The significant level was set at *p* < 0.05.

## Results

### Demographics and characteristics of the participants

**Fig. 3:**
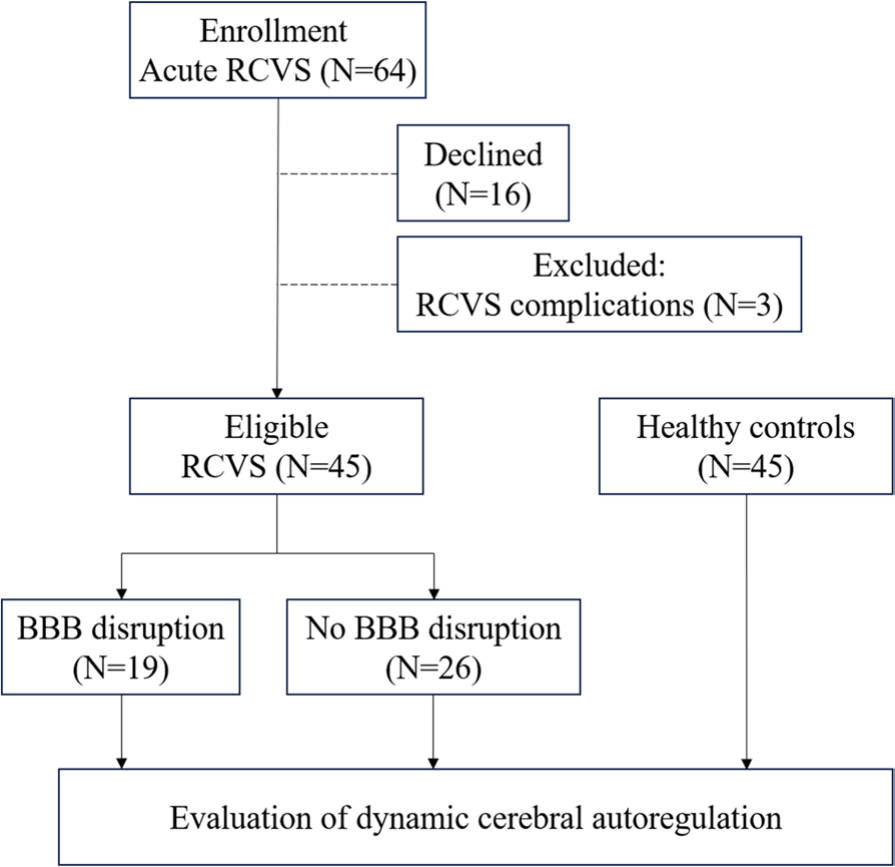
Scheme of study design and flow

During the study period, sixty-four patients diagnosed with RCVS were approached. Sixteen of them refused to participate. Three of them were excluded due to the presence of complications: one was complicated with ischemic stroke, and two were complicated with convexity subarachnoid hemorrhage. Eventually, a total of 45 patients with RCVS (41.9 ± 9.8 years old, 16 males) were enrolled, and 45 HCs (41.4 ± 12.5 years old, 16 males) were recruited in the study. There were no significant differences in age and sex (*p* > 0.05) between RCVS patients and HCs. Among the 45 patients with RCVS, 19 of them (42.2%) showed image-visualized BBB disruption on CE-FLAIR imaging. The scheme of study design was summarized in Fig. 3. Table 1 shows the demographics and the headache profiles of the RCVS subjects. There were five patients presented with potential secondary causes for RCVS: two were on illicit, vasoactive drugs (ketamine, 3,4-methylenedioxymethamphetamine, and marijuana), one was in postpartum status, one was on female reproductive hormonal treatment, and one presented with acute physical illness (acute food poisoning with violent vomiting) preceding RCVS onset. The patient in postpartum status revealed BBB disruption. The mean interval between MRI scan and dCA evaluation were 3.6 ± 3.3 days, without significant differences between patient with or without BBB disruption (3.3 ± 3.3 vs. 3.7 ± 3.4 days, *p* = 0.789). In RCVS, those with BBB disruption were older (46.3 ± 9.9 vs. 38.7 ± 8.5 years, *p* = 0.009) and with higher proportions of menopause and migraine.

**Table 1:** The demographics and the headache profiles of RCVS patients

	RCVS	BBB disruption	No BBB disruption	*p*
N	45	19	26	-
Age (yr), mean ± SD	41.9 ± 9.8	46.3 ± 9.9	38.7 ± 8.5	0.009^*^
male, n (%)	16 (36)	6 (32)	10 (38)	0.634
Hypertension, n (%)	5 (11)	4 (21)	1 (4)	0.146^a^
migraine, n (%)	13 (29)	9 (47)	4 (15)	0.043^*a^
menopause, n (%), female only	12 (41)	10 (77)	2 (13)	0.001^*a^
smoking, n (%)	16 (36)	7 (37)	9 (35)	0.878
BP surge, n (%)	5 (11)	3 (16)	2 (8)	0.636^a^
potential secondary cause, n (%)	5 (11)	1 (5)	4 (15)	0.378^a^
**Headache triggers**				
bathing/showering, n (%)	10 (22)	4 (21)	6 (23)	1.000^a^
exertion, n (%)	17 (38)	9 (47)	8 (31)	0.257
Valsalva, n (%)	27 (60)	14 (74)	13 (50)	0.109
emotion, n (%)	11 (24)	5 (26)	6 (23)	0.803
sexual activity, n (%)	22 (49)	8 (42)	14 (54)	0.436

*Data presented as mean ± standard deviation.* ^a^ *calculated with Fisher’s exact test.* ^*^ *p < 0.05*

*Potential secondary causes: exposure to illicit and vasoactive agents, postpartum status, exposure to female reproductive hormonal treatment, and acute food poisoning with violent vomiting preceding RCVS onset*

*BBB *Blood-brain barrier*, BP *Blood pressure*, RCVS *Reversible cerebral vasoconstriction syndrome

We consecutively approached 64 patients with RCVS, and enrolled 45 patients eventually (responder rate: 70.3%). Age- and sex-matched HCs were recruited. Both RCVS and HCs received evaluation for dynamic cerebral autoregulation. RCVS additionally received isotropic 3D contrast-enhanced T2-FLAIR sequencing to detect the presence of BBB disruption.

### Impaired dynamic cerebral autoregulation in RCVS

Patients with RCVS showed significantly worse dCA compared to HC indicated by larger gain in VLF band and higher Mx. Table 2 summarizes the results of dCA in RCVS and HCs.

**Table 2:** Dynamic cerebral autoregulation in HC and RCVS

parameter	frequency band	side	RCVS	HC	***p***
Gain (%/%)	VLF	L	1.6 ± 0.7	1.1 ± 0.4	0.001^*a,b^
		R	1.5 ± 0.7		0.003^*a,b^
	LF	L	1.6 ± 1.2	1.3 ± 0.5	0.061^a,^
		R	1.5 ± 0.7		0.120^a,^
Phase shift (°)	VLF	L	53.7 ± 26.4	58.0 ± 19.3	0.378
		R	50.2 ± 26.4		0.115
	LF	L	30.8 ± 32.0	37.5 ± 26.2	0.287
		R	26.2 ± 20.5		0.025^*c^
Mx	N/A	L	0.39 ± 0.20	0.31 ± 0.17	0.040^*c^
		R	0.40 ± 0.18		0.017^*b^

*Data* *presented as mean ± standard deviation.* ^*^ *p < 0.05.* ^a^ *calculated with Mann-Whitney U tests.* ^b^ *assessed by Cohen’s d showed medium to large effect.* ^c^ *assessed by Cohen’s d showed small to medium effect size*

*HC H*ealthy controls*, L *Left*, LF *Low frequency*, Mx *Mean flow correlation index*, N/A *Not applicable*, R *Right*, RCVS *Reversible cerebral vasoconstriction syndrome*, VLF *Very-low frequency

### Association between BBB disruption and cerebral dysautoregulation in RCVS

**Fig. 4:**
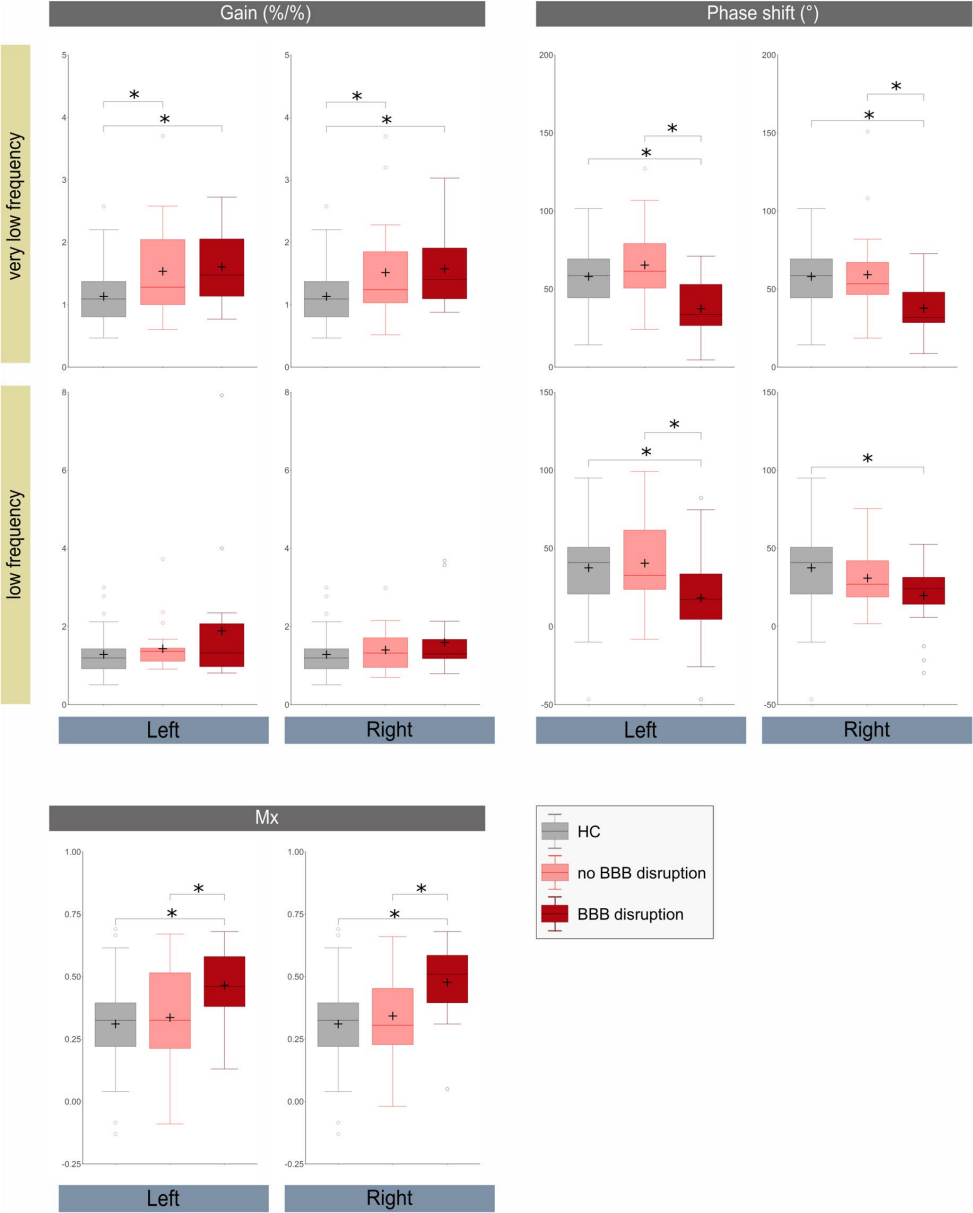
Comparison of Gain, Phase Shift, and Mean Flow Correlation Index Between Healthy Controls, RCVS Patients Without BBB Disruption, and RCVS Patients with BBB Disruption. * *p*  < 0.05; The horizontal lines within the box indicate the median, and the + indicates the mean. The error bars indicate the 1.5 interquartile range of the lower and upper quartiles. The open circle indicates outliers

When taking BBB disruption into consideration, we found a significant difference of dCA between RCVS patients with and without BBB disruption, as well as between RCVS patients and HCs. By having the smallest VLF phase shift and largest Mx, RCVS with image-proven BBB disruption showed the worst dCA as compared to RCVS without BBB disruption and to HC. The RCVS groups (either with or without BBB disruption) had larger VLF gain as compared to HCs, suggesting worse dCA in RCVS. Table 3; Fig. 4 summarize the comparisons between the three groups regarding dCA. Univariate logistic regression models demonstrated the association between BBB disruption and dCA metrics, as well as clinical features, namely, age and having a history of migraine. Older age, presence of a history of migraine, having less VLF phase shift (bilateral), having less LF phase shift (left), as well as larger Mx (bilateral) increased the odds of BBB disruption. Five different multivariable models examined the association between each dCA metrics and BBB disruption after controlling for age and history of migraine. VLF phase shift on both sides remained to be associated with BBB disruption (Nagelkerke R^2^: 53.8% on the left, 51.2% on the right). The results were summarized in the Table 4.

**Table 3:** BBB disruption and cerebral dysautoregulation in RCVS

parameter	frequency band	side	BBB disruption	No BBB disruption	HC	ANOVA ***p***
Gain (%/%)	VLF	L	1.6 ± 0.6	1.5 ± 0.7	1.1 ± 0.4	0.004^*a,b^
		R	1.6 ± 0.6	1.5 ± 0.8		0.011^*a,b^
	LF	L	1.9 ± 1.7	1.4 ± 0.6	1.3 ± 0.5	0.170^a^
		R	1.6 ± 0.8	1.4 ± 0.6		0.221^a^
Phase shift (°)	VLF	L	37.5 ± 18.1	65.3 ± 25.5	58 ± 19.3	< 0.001^*b^
		R	37.7 ± 17.9	59.2 ± 28.1		0.003^*b^
	LF	L	18.2 ± 31.5	40.4 ± 29.6	37.5 ± 26.2	0.023^*b^
		R	19.7 ± 21.8	30.9 ± 18.5		0.024^*b^
Mx	N/A	L	0.46 ± 0.14	0.34 ± 0.22	0.31 ± 0.17	0.008^*b^
		R	0.48 ± 0.15	0.34 ± 0.18		0.002^*b^

*Data presented as mean ± standard deviation.* ^*^ *p < 0.05.* ^a^ *calculated with Kruskal-Wallis tests.* ^b^ *Assessed by Cohen’s f showed medium to large effect size*

*BBB *Blood-brain barrier*, HC *Healthy control*, L *Left*, *LF Low frequency*, Mx Mean flow correlation index, N/A *Not applicable*, R *Right*, RCVS *Reversible cerebral vasoconstriction syndrome*, VLF *Very-low frequency

**Table 4:** The results of univariate and multivariable Logistic regression model demonstrating the association of clinical features and dCA metrics with BBB disruption

	univariate		Models controlling age and migraine^a^	
	*p*	OR (95% CI)	*p*	OR (95% CI)
age	0.014^*^	1.10 (1.02 ~ 1.18)		
migraine	0.025^*^	4.95 (1.23 ~ 19.97)		
VLF phase shift				
Left	0.003^*^	0.94 (0.90 ~ 0.98)	0.024^*^	0.95 (0.91 ~ 0.99)
Right	0.012^*^	0.95 (0.92 ~ 0.99)	0.031^*^	0.95 (0.91 ~ 0.995)
LF phase shift				
Left	0.032^*^	0.98 (0.95 ~ 0.99)	0.135	0.98 (0.95 ~ 1.01)
Right	0.083	0.97 (0.94 ~ 1.004)		
Mx				
Left	0.037^*^	48.26 (1.26 ~ 1850.35)	0.194	33.171 (0.17 ~ 6519.24)
Right	0.019^*^	140.90 (2.28 ~ 8689.69)	0.051	104.42 (0.98 ~ 11156.96)

*p < 0.05. ^a^ *The column summarized the results of five different models examined the association between each dCA metrics and BBB disruption after controlling for age and history of migraine*

*OR *Odds ratio*, CI *Confident interval*, VLF *Very-low frequency*, LF *Low frequency*, Mx *Mean flow correlation index

## Discussion

In the current investigation, we have substantiated that patients with RCVS had poorer dCA as compared to HCs. Moreover, we have delineated the association between BBB disruption and impaired dCA, revealing that RCVS patients with BBB disruption had worse dCA, as compared to RCVS patients without discernible BBB disruption. To our best knowledge, this is a novel finding that provides the direct evidence and mechanistic insights of dCA dysfunction in RCVS and its association with image-proven BBB disruption.

TCHs in RCVS can be provoked by Valsalva-like maneuvers including coughing, straining, physical exertion, sexual activity or singing [3, 6]. These triggers are related to rapid BP changes in the span of minutes or even seconds. Therefore, the dCA, which measures the instantaneous changes of CBF in response to the fluctuating BP, is a more rational means to study the autoregulation in RCVS and its pathophysiology. In fact, BP surge has been identified to be a risk factor of BBB disruption in RCVS [14]. Corroborated with this finding, we noticed that patients with RCVS and BBB disruption exhibited worse dCA, particularly in VLF bands after adjusting potential confounders. It has been reported that VLF cerebral hemodynamic oscillation implies vasomotor activities derived from the large arterioles (50–100 μm), which receives intrinsic neurogenic innervation from brainstem nuclei and local interneurons [35–38]. Our results suggested that in RCVS, vasomotor activities were altered, probably mediated through dysfunctional neurovascular coupling.

In the present RCVS cohort, patients with image-proven BBB disruption were significantly older than those without. This finding was in line with our experience that contrast-medium extravasation was more likely to be observed in older patients with RCVS [14], suggesting the heterogenicity in RCVS population. We speculate that either the age-dependent loss of BBB integrity [39, 40] may play a role, or the self-protective mechanisms that prevents BBB from the damage of excessive pulsatile flow may be compromised in the aging process. However, most studies indicated that dCA was unaffected by healthy aging in normal population [41–43]. An alternative explanation is that older patients may be affected by arterial stiffness or reduced baroreflex sensitivity that compromise the protective mechanism from excessive pulsatile flow. Therefore, the age differences between two groups may be an intrinsic factor that contributes to the disease pathogenesis.

The proportion of menopause was higher in patients with BBB disruption. The permeability of BBB was vigorously influenced by estrogen and progestogen [44]. Study directly assessed dCA in pre- and postmenopausal women is vacant [45]. However, there was evidence indicating shear-mediated dilation of the ICA, a potential surrogate for cerebrovascular endothelial function, was reduced in postmenopausal women [46]. The phenomenon was associated with lower serum estradiol level, independent of age. Epidemiology showed that RCVS predominantly occurred in middle-aged women who are around perimenopause [1]. Post-partum angiopathy, one of the former names of RCVS, also links RCVS to dramatical fluctuation of sex hormones. It is rational to postulate that sex hormones also play a role in RCVS pathogenesis, mediating with both altered BBB integrity and cerebral autoregulation. We also found that patients with RCVS complicated with BBB disruption are more likely to have a history of migraine. However, a recent meta-analysis found no significant differences in phase shift, gain, and Mx of dCA between patients with migraine and controls [47]. Hence, more studies are warranted to verify the association between BBB disruption and migraine comorbidity in RCVS.

Some previous studies have provided supportive findings that suggest impaired cerebral autoregulation in patients with RCVS. In a retrospective analysis, Topcuoglu et al. [48] discovered severely decreased breath holding index (BHI) during breath-holding test in ten patients with RCVS, indicating exhausted cerebral vasomotor reactivity that normally responds to hypercapnia through cerebral endothelium-dependent vasodilation. Later, Choi et al. [49] conducted a study with similar methodology in twenty-eight patients with RCVS, and the results were compatible. Besides, part of RCVS patients (10/28, 35.7%) repeated breath-holding test at three-month follow-up. Three out of ten failed to show BHI normalization. Our research [29] focused on the dynamic temporal evolution of white matter hyperintensity lesions (WMHs) in RCVS proposed that the WMHs in RCVS may be resulted from the cerebral dysautoregulation under excessive central pulsatile flow and damaging of brain microvascular structure and neurovascular unit. A recent study [50] of our group discovered that a panel of circulating micro RNA (miRNA) that targets *END1 (*endothelin-1), the gene which is responsible for cerebral vascular tone, could differentiate RCVS patients in acute stage and controls. Of them, miR-130a-3p was associated with image-proven BBB disruption, and its overexpression leads to increased permeability in in vitro human BBB model. By direct measuring of dCA in patients with RCVS and establishing the association between impaired dCA and image-proven BBB disruption, the present study reinforces the connections between impaired autoregulation and BBB disruption in the pathogenesis of RCVS.

The strengths of this study include a RCVS cohort that recognized by experienced headache specialists with validated criteria and a well-trained research group who conducted the experiments with structured protocols, including high-resolution images obtained from the same high-field MRI machine and an established, standardized system of the dCA measurement. However, our study has limitations. Limited to study design, we are unable to clarify the causality between cerebral dysautoregulation and BBB disruption. Given the low incidence of RCVS, it is impractical and virtually impossible to prospectively measure dCA *before* an individual being diagnosed with RCVS to determine whether impaired dCA is causally related to RCVS occurrence and BBB disruption. Second, some patients with RCVS had hypertension, while most HC denied a history of hypertension. There is a widespread myth that hypertension leads to impaired dCA. The concept had been proven incorrect by plenty of studies [19, 51–54] that revealed intact dCA in young, middle-aged, and senile participants with hypertension. A group of age- and sex-match HCs, as in this study, is well-fit to generate unbiased results.

## Conclusions

We demonstrate the dysfunction of dCA in patients with RCVS, manifesting as unstable CBF under BP alterations, particularly in those with imaging-proven BBB disruption. These novel findings suggest that cerebral dysautoregulation plays a pivotal role in RCVS pathophysiology and may be relevant to complications associated with BBB disruption.

## Data Availability

All the data presented in this study are available from the corresponding authors on reasonable requests.

## Abbreviations

3D: 3-dimensional
BBB: Blood-brain barrier
BHI: Breath holding index
BP: Blood pressure
CBF: Cerebral blood flow
CBFV: Cerebral blood flow velocity
CE-FLAIR: Contrast-enhanced fluid-attenuated inversion recovery imaging
CPP: Cerebral perfusion pressure
CTA: Computed tomography angiography
dCA: Dynamic cerebral autoregulation
Gd: Gadolinium
HCs: Healthy controls
ICA: Internal carotid artery
ICHD-3: International Classification of Headache Disorders, third edition
LF: Low frequency
LSD: Least significant difference
MCA: Middle cerebral artery
miRNA: Micro RNA
MRA: Magnetic resonance angiography
Mx: Mean flow correlation index
RCVS: Reversible cerebral vasoconstriction syndrome
T1WI: T1-weighted imaging
TCHs: Thunderclap headaches
TFA: Transfer function analysis
VLF: Very-low frequency
WMHs: White matter hyperintensity lesions
